# Clinical manifestations of delayed reaction following mass hornet envenomation: a case report

**DOI:** 10.1097/MS9.0000000000000453

**Published:** 2023-04-07

**Authors:** Urza Bhattarai, Anil Acharya, Ram Sharan Shrestha, Arun Gautam, Ayush Anand, Srista Manandhar, Sanjib Kumar Sharma

**Affiliations:** a Department of Internal Medicine, B.P. Koirala Institute of Health Sciences; b B.P. Koirala Institute of Health Sciences, Dharan, Nepal

**Keywords:** acute kidney injury, case reports, delayed reaction, hornet stings

## Abstract

Delayed reaction following mass hornet envenomation is associated with various clinical manifestations.

**Case Presentation:**

The authors present a case of a 24-year-old male from eastern Nepal, who presented following mass envenomation by hornet stings. He had progressive yellowish discoloration of skin and sclera, myalgia, fever, and dizziness. He had passage of tea-coloured urine followed by anuria. Laboratory investigations suggested acute kidney injury, rhabdomyolysis, and acute liver injury. The authors managed the patient with supportive measures and haemodialysis. There was complete recovery of liver and renal function in the patient.

**Discussion:**

The findings in this patient were similar to other cases reported in the literature. These patients must be managed supportively, with few requiring renal replacement therapy. Most of these patients recover completely. In low-middle-income countries like Nepal, delay in seeking care and delay in reaching care is associated with severe clinical manifestations. Delayed presentation can lead to renal shutdown and mortality; hence, early intervention is simple, and, crucial.

**Conclusion:**

This case highlights the occurrence of delayed reaction following mass envenomation by hornets. Also, the authors show an approach to managing such patients, similar to managing any other case with acute kidney injury. In these cases, an early simple intervention can prevent mortality. It is crucial to train healthcare workers regarding toxin induced acute kidney injury and the importance of early identification and intervention.

## Introduction

Highlights

- Clinical consequences following mass hornet stings ranges from anaphylactic reaction to multi organ failure and death.
- We report a case of a 24-year-old male from Nepal with delayed presentation to hospital setting associated with organ failure in the form of acute kidney injury. Although usually self-limiting, hornet envenomation can lead to mortality, particularly in a setting with mass envenomation and delayed presentation.
- We describe the clinical manifestations, natural history, laboratory parameters, and approach to management of a patient with hornet envenomation.
- An early simple intervention, such as hydration can prevent acute kidney injury and its consequences. It is crucial to train healthcare workers regarding toxin induced acute kidney injury and the importance of early intervention.

Hornets belong to the family of Vespidae of the order Hymenoptera^1^. Intense pain at the site of the sting is attributed to serotonin, waspkinins, and acetylcholine^1^,^2^. The hornet venom contains antigen 5, a major allergen leading to immediate anaphylactic reactions^1^. These reactions can be local, regional, or systemic^1^,^3^. A delayed response to envenomation can manifest as serum sickness, glomerulonephritis, coagulation abnormalities, acute hepatitis, or arthritis^1^,^3^. Many cases of acute kidney injury following hornet sting have been reported^4^. Although usually self-limiting, it can also lead to mortality, particularly in a setting with mass envenomation and delayed presentation. The lethality depends also on the species of the stinging insects^1^,^5^–^9^. Herein, we report the case of a 24-year-old male from Nepal with delayed presentation of acute kidney injury and liver injury following mass hornet envenomation. The case has been reported in line with the SCARE 2020 guidelines^10^.

## Case report

**Figure 1:**
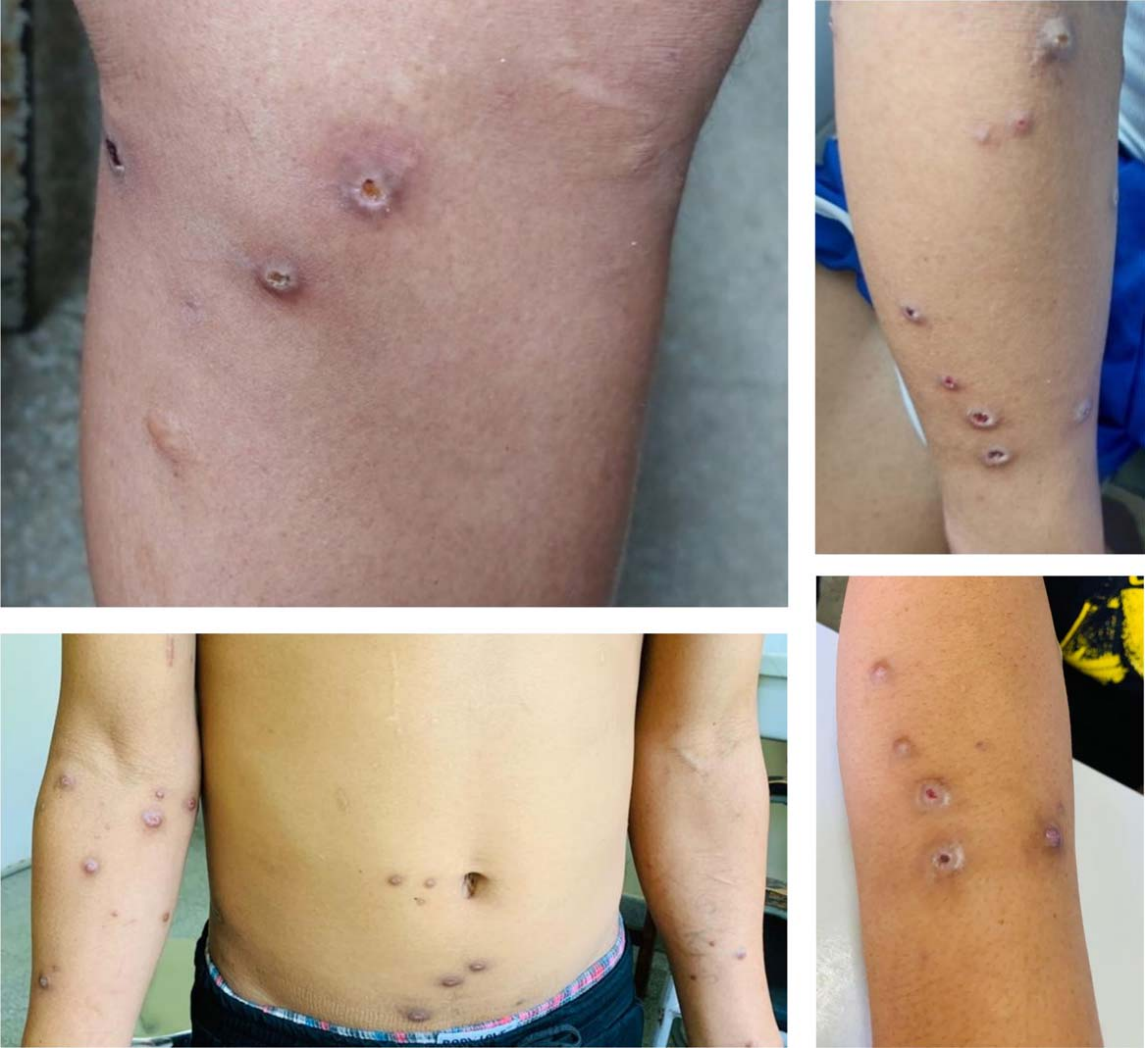
Multiple hornet sting lesions over torso and extremities.

A 24-year-old male, from eastern Nepal, with no prior co-morbidities, presented following mass envenomation by hornet stings 36 h ago. Initially, local redness, pruritis, and pain developed at the sting site within 30 min. For this, the patient took some over-the-counter medications for pain and pruritus relief. There was no history of previous hornet stings and immediate severe manifestations. The next day he developed gradually progressive yellowish discoloration of skin and sclera, myalgia, fever, and dizziness. It was associated with passage of reddish tea-coloured urine followed by decreased urine output. On presentation, urinary catheterization was done, and urine output was monitored, which came out to be less than 50 ml in 24 h. There was no history of loss of consciousness, chest pain, abdominal pain, headache, limb weakness, or palpitation. On examination, he had icterus. Multiple hornet stings, thirty-two in number (Fig. 1) were visible in the patient’s torso and upper and lower extremities. His height was 157 cm and weight of 62 kgs, body mass index of 25.1 kg/m^2^. His vitals were stable, and systemic examination was unremarkable.

Laboratory parameters (Table 1) showed features suggestive of acute hepatitis and acute kidney injury with hyperkalemia. Raised creatine phosphokinase with hyperuricemia was suggestive of rhabdomyolysis. Also, he had leucocytosis (total leucocyte count: 30,000 cells/mm^3^). His urinalysis revealed 3 + proteinuria, 1–2 pus cells, and 0–2 red blood cells. Serum amylase and lipase were 181 and 421 U/l, respectively. Ultrasonography of the abdomen and pelvis showed enlarged bilateral kidneys with increased echogenicity with compression of renal sinuses and mild ascites.

**Table 1:** Laboratory parameters of the patient.

Lab parameters	Day 1	Day 14 (discharge)	Day 30 (follow-up)	Reference
Aspartate aminotransferase (U/l)	3307	78	42	09–43
Alanine aminotransferase (U/l)	1130	64	32	10–35
Total bilirubin (mg/dl)	20.9	1.23	1.0	0.2–1.2
Conjugated bilirubin (mg/dl)	11.04	1.0	0.1	0.0–0.2
Urea (mg/dl)	189.9	49	55	10–50
Creatinine (mg/dl)	8.5	5.9	1.3	0.3–1.2
Sodium (mmol/l)	131	133	135	136–145
Potassium (mmol/l)	6.4	4.4	4.0	3.5–5.0
Creatinine phosphokinase (U/l)	10147			<167
Serum uric acid (mg/dl)	11.0			2.5–6.8

The patient was managed under the supervision of a nephrologist and internists. We diagnosed acute kidney injury, acute hepatitis, and hyperkalemia secondary to mass hornet envenomation. The patient underwent emergency haemodialysis for anuria with refractory hyperkalemia. He was managed with intravenous fluids, antipyretics, and alternate-day haemodialysis because of persistent anuria. He underwent seven haemodialysis sessions in 2 weeks for anuria (Table 2). The first haemodialysis session was of 2 h while rest of the sessions were of 3 h and 30 min each. His urine output was persistently less than 100 ml in the first week. During the second week, urine output gradually improved. At 2 weeks, urine output doubled to 200 ml in 24 h and improved to 400 ml in the next 24 h. At discharge, he was advised adequate hydration with twice-a-week haemodialysis until normalization of renal function. After 1 month’s follow-up, he had normal urine output with normal renal function.

**Table 2:** Renal parameters of the patient.

Lab parameters	Day 1	Day 3	Day 5	Day 7	Day 9	Day 11	Day 13	Reference
Urea (mg/dl)	189.9	152	168.3	144	93.1	73.5	76.9	10–50
Creatinine (mg/dl)	8.5	7	7.5	7.6	6.0	6.9	7.6	0.3–1.2
Sodium (mmol/l)	131	135	134	134	135	134	134	136–145
Potassium (mmol/l)	6.4	4.5	4.2	4.0	3.3	3.7	4.3	3.5–5.0

## Discussion

Hornet envenomation can lead to various types of reactions. The patients can have pain, pruritis, and a burning sensation at the sting site, usually self-limiting within hours^1^,^3^. It can form papules and pustules, which disappear in a few weeks^1^. Secondary skin infections such as cellulitis and necrotizing fasciitis can lead to sepsis^3^. Some patients have regional reactions, which may lead to vascular insufficiency in the involved areas^1^. In some instances, severe life-threatening anaphylactic reactions can occur, which may lead to significant mortality^1^,^3^,^7^,^9^,^11^.

Some experimental evidences in animal models, as reported in Schmidt *et al*.^5^ have shown that an average number of stings for a lethal dose based on four species of hornets would be about 100 stings per 50 kg person, but the actual number varied substantially depending upon the species of hornet. Sakhuja *et al*.^12^ reported acute kidney injury due to acute tubular necrosis, with few patients having features suggestive of rhabdomyolysis. Also, patients had elevated alanine aminotransferase and aspartate aminotransferase^12^. Chugh *et al*.^13^ also reported acute tubular necrosis following multiple hornet stings, confirmed by renal biopsy and attributed to intravascular haemolysis. A case series by Xuan *et al*.^9^ of 65 cases of mass envenomation by hornets revealed that approximately three-fifths of patients had AKI, with half of the patients requiring haemodialysis. The majority of patients had rhabdomyolysis, and those who presented late had an increased risk of AKI^9^. Also, the patients required an average of 3.9 dialysis sessions, with complete recovery in most patients^9^. Reports from Nepal have also reported these patients with acute kidney injury and acute hepatitis, with some patients requiring haemodialysis^14^,^15^,^16^.

In our patient, initially, there was localized pain. Pain following a hornet sting can be attributed to serotonin, waspkinins, and acetylcholine in hornet venom^1^,^2^. Later the patient had icterus, anuria, deranged liver function test, and renal function test following mass envenomation by hornets. Also, the patient has raised creatine phosphokinase, suggestive of rhabdomyolysis. These findings suggested a diagnosis of acute kidney injury and acute hepatitis secondary to rhabdomyolysis due to mass envenomation by hornet stings^4^,^12^,^13^,^15^,^17^. The mainstay of management of AKI is supportive management with adequate hydration and renal replacement therapy when indicated^17^. Our patient presented early and required seven haemodialysis sessions with complete recovery of renal function.

Before, presenting to the hospital, the patient took over-the-counter medications for symptomatic treatment without seeking medical help. Delay in seeking care and reaching care can lead to significant mortality. Also, healthcare workers in rural areas are not aware of the approach to management of these cases, where these cases are initially reported^18^. There is a need to aware and educate healthcare providers in areas where these cases are common. Hence, we want to focus that the delayed reactions to hornet stings are actually quite common and almost a norm for cases in which mass venomation has occurred. One of the problems with treating such patients is that they go home after several hours feeling relatively good and then come back 1–3 days later as their kidneys are failing and their health is in serious trouble. Therefore, in such a situation the patient needs to be monitored continuously for up to 4 days, especially if several dozen or more hornet stings have been received.

## Conclusion

Our case highlights the occurrence of delayed reaction leading to acute kidney injury following mass envenomation by hornets. Also, we show an approach to managing such patients, similar to managing any other case with acute kidney injury. In these cases, an early simple intervention, such as hydration can prevent acute kidney injury and its consequences. It is crucial to train healthcare workers regarding toxin induced acute kidney injury and the importance of early identification and intervention.

## Ethical approval

Ethical approval was not required for this case report.

## Consent

Written informed consent was obtained from the patient for publication of this case report and accompanying images. A copy of the written consent is available for review by the Editor-in-Chief of this journal on request.

## Sources of funding

The authors did not receive any funding for this manuscript.

## Author contribution

U.B., A.A., R.S.S., A.G., and S.K.S. were involved in management of the case and have made a substantial contribution to the concept of the article. U.B., A.A., S.M., A.G., and S.K.S. drafted the article. A.G., U.B., and S.K.S. were involved in critically revising the manuscript.

## Conflicts of interest disclosure

The author(s) have no conflict of interest to declare.

## Registration of research studies

1. Name of the registry: NA.
2. Unique identifying number or registration ID: NA.
3. Hyperlink to your specific registration (must be publicly accessible and will be checked): NA.

## Guarantor

Sanjib Kumar Sharma.

## Provenance and peer review

Not commissioned, externally peer-reviewed.
